# Flap Necrosis after Palatoplasty in Patients with Cleft Palate

**DOI:** 10.1155/2015/516375

**Published:** 2015-07-26

**Authors:** Percy Rossell-Perry

**Affiliations:** ^1^Post Graduate Studies, School of Medicine, San Martin de Porres University, Lima, Peru; ^2^“Outreach Surgical Center Lima PERU” ReSurge International, Schell Street No. 120 Apartment 1503 Miraflores, Lima, Peru

## Abstract

Palatal necrosis after palatoplasty in patients with cleft palate is a rare but significant problem encountered by any cleft surgeon. Few studies have addressed this disastrous complication and the prevalence of this problem remains unknown. Failure of a palatal flap may be attributed to different factors like kinking or section of the pedicle, anatomical variations, tension, vascular thrombosis, type of cleft, used surgical technique, surgeon's experience, infection, and malnutrition. Palatal flap necrosis can be prevented through identification of the risk factors and a careful surgical planning should be done before any palatoplasty. Management of severe fistulas observed as a consequence of palatal flap necrosis is a big challenge for any cleft surgeon. Different techniques as facial artery flaps, tongue flaps, and microvascular flaps have been described with this purpose. This review article discusses the current status of this serious complication in patients with cleft palate.

## 1. Background

Severe complications in patients after cleft palate surgery are not common.

**Figure 1:**
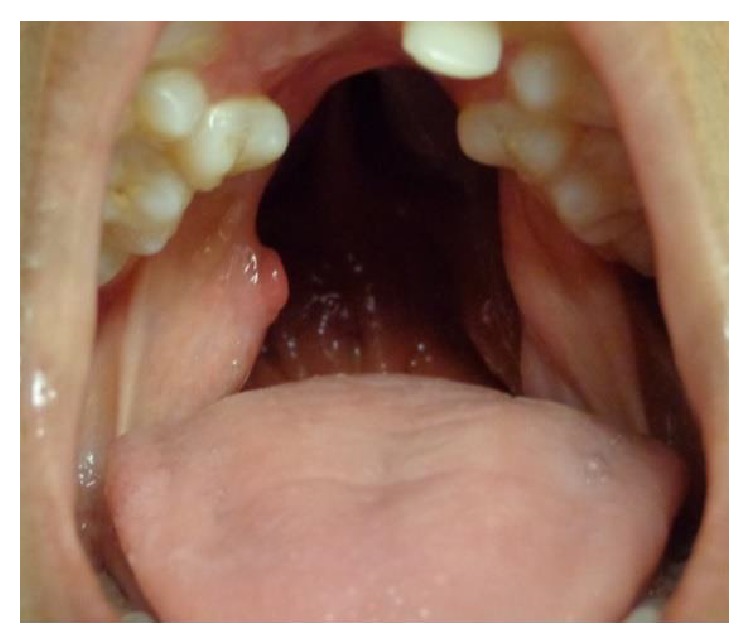
Patient with bilateral cleft palate and large palatal defect after mucoperiosteal flap necrosis. The extent of the defect is bigger than the original cleft size.

**Figure 2:**
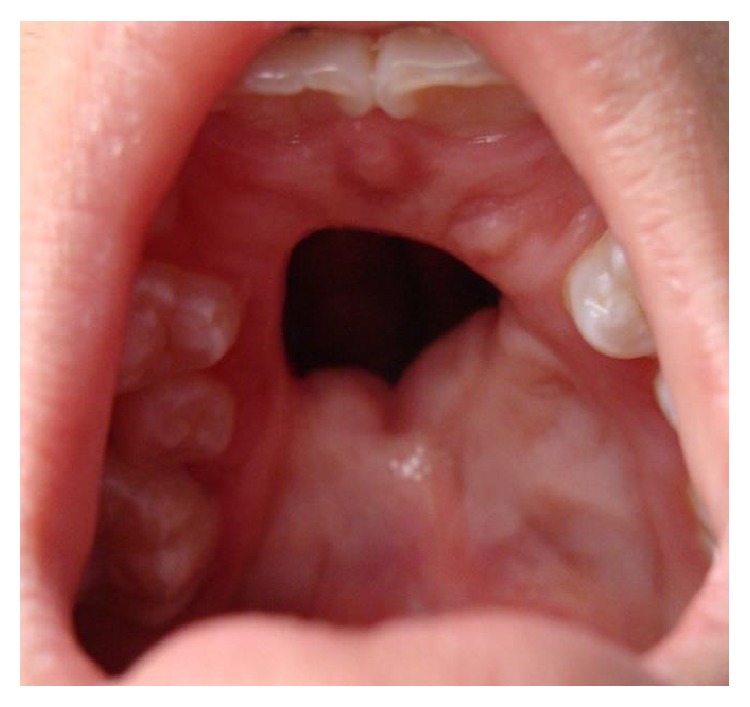
Patient with isolated cleft palate and severe fistula after mucoperiosteal flap necrosis.

Severe defects are characterized by extended deficiency of tissues usually wider than the primary cleft and presented as severe fistulas or absence of palatal tissue in worst cases (Figures 1 and 2).

These defects are commonly in relation with loss of palatal tissue after palatal flap necrosis. The extent of functional impairment is great and they have psychological, social, and developmental consequences.

This condition permits a free flow of food into the nasal cavity in a volume large enough that it may exit through the naris. In addition, the nasal secretion seeps into the mouth producing bad taste, malodorous breath, and poor oral hygiene.

Furthermore, these sequels affect speech and resonance with hypernasality, audible nasal scape, and weakness of pressure consonants.

Preservation of the mucoperiosteal flaps after palatoplasties guarantees the closure of the cleft and the functional outcomes of these surgeries (speech, feeding).

Few studies have addressed this disastrous complication and the prevalence of this problem remains unknown.

Prevalence of palatal flap necrosis in three centers in Peru was 0.34% (Table 1) [1].

Two cases were bilateral cleft palates and two incomplete cleft palates. Three of them were children and one adult.

Studied prevalence in a study made by Diah et al. from Chang Gung University of Taiwan was 64/2 (3.1%) [2].

Another study from Nigeria observed two cases of flap necrosis (1%) in patients with bilateral cleft palates [3].

A multivariate analysis made in 709 patients by Deshpande et al. found low rate of total or partial flap necrosis (less than 1%) [4].

Management of severe fistulas observed as a consequence of palatal flap necrosis is a big challenge for any cleft surgeon. Different techniques as facial artery flaps, tongue flaps, and microvascular flaps have been described with this purpose.

This review article discusses the current status of this serious complication in patients with cleft palate.

## 2. Anatomy

The palate has a rich blood supply.

**Figure 3:**
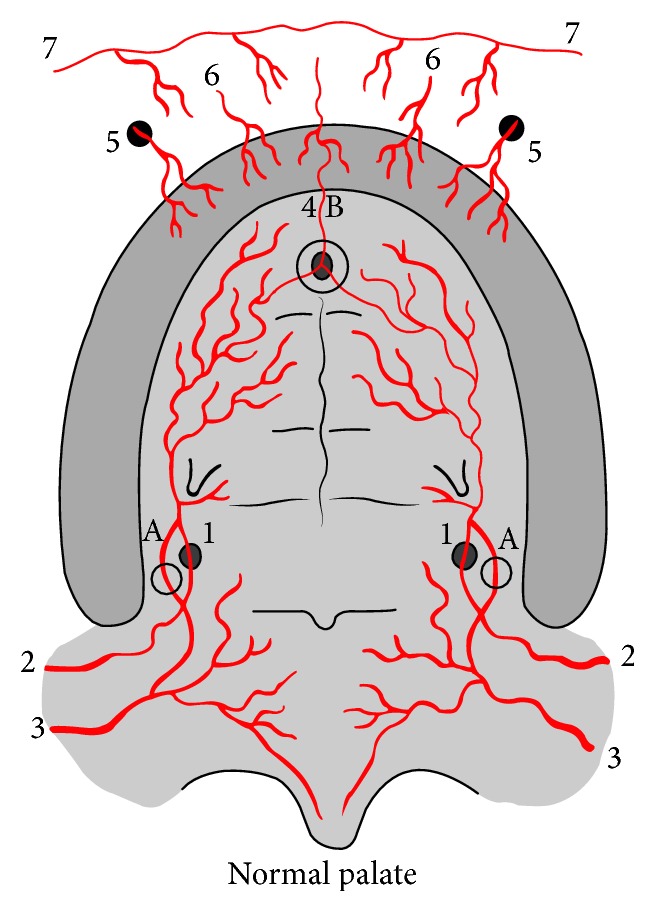
Noncleft palate vascularization. 1: greater palatine artery; 2: descending palatine artery; 3: ascending palatine artery; 4: anterior palatine artery; 5: branches from infraorbital artery; 6: superior alveolar artery; 7: branches superior labial artery. A: Vascular anastomoses between descending palatine artery and ascending palatine artery through lesser palatine vessels; B: vascular anastomoses between greater palatine artery and anterior palatine artery.

Blood supply of the palate is carried by branches of the external carotid artery: greater palatine, ascending palatine, infraorbital, alveolar, superior labial arteries, and branches of the ascending pharyngeal artery [5] (Figure 3).

Vascularization of the mucoperiosteum of the hard palate comes mainly from the greater palatine vessels (branch of descending palatine artery from the maxillary artery) which emerge from the greater palatine foramen [6, 7].

**Figure 4:**
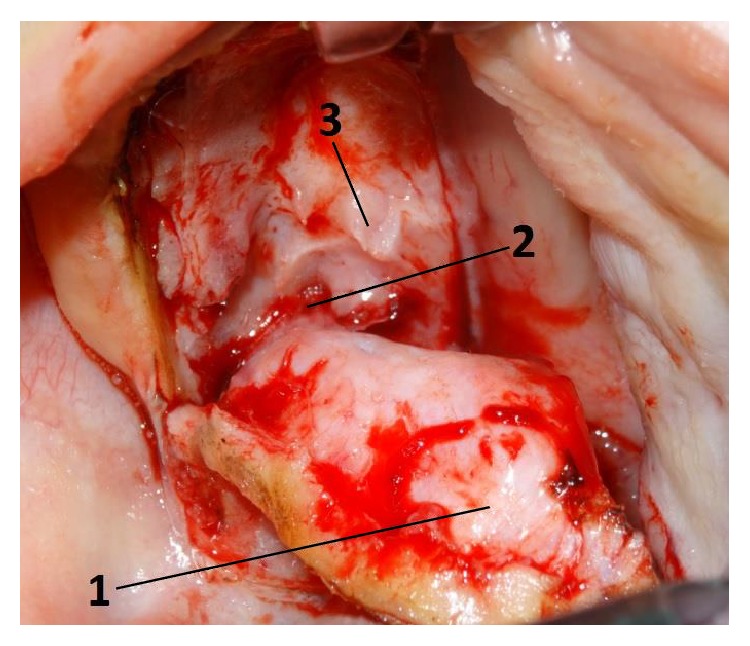
Anatomical relations of the greater palatine vessels: 1, mucoperiosteal flap; 2, greater palatine vessels coming from greater palatine foramen; 3, palatal spine.

Location of the greater palatine foramen during the surgery let us prevent the injury of this artery (Figure 4).

Main location of greater palatine foramen was located at the level of the second molar (35.7%), interproximal to the second and third molars (35.7%) in women, and at the level of the second molar in men (65%) as described by Klosek and Rungruang in 2009 [8].

A study made by Fu et al. in 2011 [9] observed that the most frequent greater palatine foramen location was between the second and third molars (66.6%) and similar results were observed by studies made in Chinese skulls by Wang et al. in 1988 [10] and Ajmani in Nigerian and Indian skulls [11].

Other important anatomical landmarks of the greater palatine foramen are distance from the foramen to the posterior border of the hard palate (approximately 3 mm), perpendicular distance of the foramen to the midline maxillary suture (about 14 mm) [12], 0.3 cm from the inner border of the alveolar ridge [13], 10.72 mm from the alveolar crest, and 4.38 mm from the surface of the palatal mucosa in the palatal region between the first premolar and the first molar [14].

Differences in relation with age were not considered in these studies since they were made in adult skulls. Therefore, application of these findings in primary cleft palate surgery is limited and should be studied.

Some ethnic variations about the greater palatine foramen have been found in different studies and the bilateral symmetry of greater palatine foramen on both sides of each skull is remarkable [11].

Another point of reference of the hard palate to locate the greater palatine artery is bony prominences named as palatine spines (Figure 4).

These are small projections that arise from the middle, posterior margin of the maxilla near their junction with the palatine bone and divide the medial and lateral grooves.

These prominences project a few millimeters over the greater palatine vessels as they pass forward on the inferior surface of the palate and can be easily identified during the cleft palate surgery (Figure 3).

Palatal spines were frequently observed as bony prominences (66.3%, 57 sides) and were located at 6.49 ± 1.76 mm from the greater palatine foramen, with a length of 10.42 ± 2.45 mm [15].

In my personal experience, palatine spines are the most important point of reference to locate the greater palatine vessels during the surgery.

These bony prominences may be absent or small in 14.7% of cases (mostly in syndromic patients) [16].

The greater palatine artery reaches the mucoperiosteum of the hard palate and runs anteriorly, in the lateral portion of the palate near its junction with the alveoli.

The artery emerged in the posterior lateral section of the greater palatine foramen and it continued its pathway into an osseous groove until it reached the retroincisive zone.

The artery is divided into two or three branches at the exit of the foramen [17].

The most common greater palatine artery branching pattern was the one which gave off the medial and canine branches after the palatal spine (41.7%) [15].

In the same study made by Klosek and Rungruang, they observed that the greater palatine artery was branching most frequently at the level of first premolar (38%) and at first and second molars together (43%) in women [8].

In cleft patients, additional vascularization is provided by multiple branches passing both medially from the nasal mucosa and laterally toward the alveolus [18].

There are numerous arterial connections between nasal and palatal mucosa with connections made at bony margins and by perforating osseous arteries [18, 19].

These branches are divided by medial and lateral incisions and subperiosteal dissection during conventional palatoplasty.

The descending palatine artery provides additional branches, named as lesser palatine arteries, which enters the palate through the lesser palatine foramen to supply the soft palate [19, 20] (Figure 1).

The soft palate is supplied by the following arteries: (a) ascending palatine artery (from facial artery mainly), (b) tonsillar (branch of the ascending palatine artery), (c) ascending pharyngeal artery (from external carotid artery), (d) lesser palatine arteries (from the greater palatine artery), and (e) recurrent pharyngeal artery (from the external carotid artery) [18, 21].

**Figure 5:**
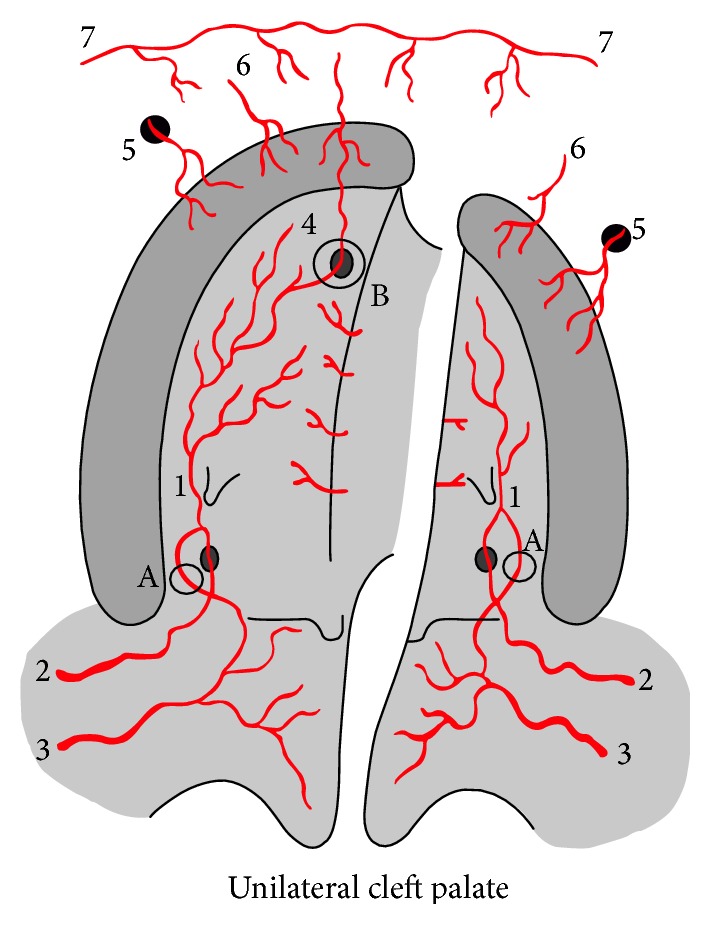
Unilateral cleft palate vascularization. 1: greater palatine artery; 2: descending palatine artery; 3: ascending palatine artery; 4: anterior palatine artery; 5: branches from infraorbital artery; 6: superior alveolar artery; 7: branches superior labial artery. A: vascular anastomoses between descending palatine artery and ascending palatine artery through lesser palatine vessels; B: vascular anastomoses between greater palatine artery and anterior palatine artery.

**Figure 6:**
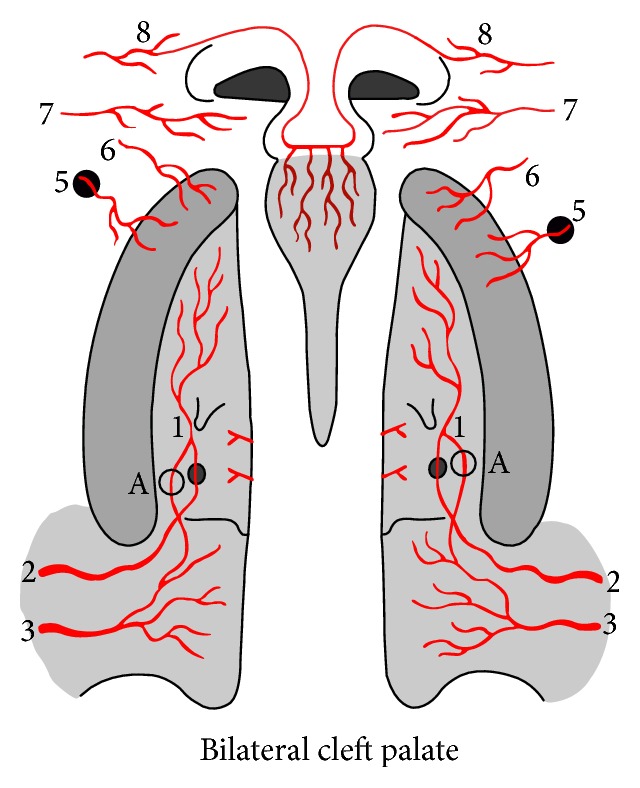
Bilateral cleft palate vascularization. 1: greater palatine artery; 2: descending palatine artery; 3: ascending palatine artery; 5: branches from infraorbital artery; 6: superior alveolar artery; 7: branches superior labial artery; 8: dorsal nasal artery. A: vascular anastomoses between descending palatine artery and ascending palatine artery through lesser palatine vessels.

**Figure 7:**
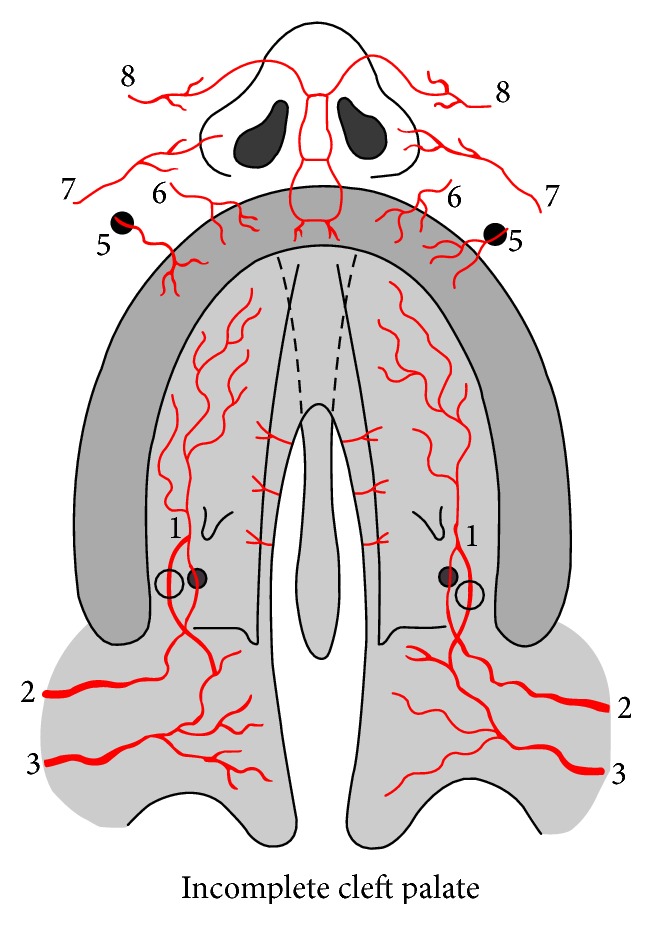
Incomplete cleft palate vascularization. 1: greater palatine artery; 2: descending palatine artery; 3: ascending palatine artery; 5: branches from infraorbital artery; 6: superior alveolar artery; 7: branches superior labial artery; 8: dorsal nasal artery. A: vascular anastomoses between descending palatine artery and ascending palatine artery through lesser palatine vessels.

Facial artery provides additional blood supply to the hard palate by the ascending palatine artery (Figures 3 to 7).

This artery supplies mainly the superior pharyngeal constrictor and the soft palate.

There is a large network of anastomoses between the vessels that supply the hard palate and soft palate [21] (Figures 3 to 7).

The most important are the anastomoses between the ascending palatine and lesser palatine arteries and acquire importance when the greater palatine artery is sectioned accidentally during palatoplasty.

Few studies have been published reporting anatomical variation of these vessels and its relation with some nondesirable outcomes after cleft palate repair.

Maher in 1977 described position and variations of the arteries of the palate in cleft patients and observed in cleft and noncleft fetuses arterial anastomoses between the greater palatine artery and infraorbital, superior alveolar, sphenopalatine branches from the maxillary artery, and superior labial branches from the facial artery [5, 18] (Figures 5, 6, and 7).

Gauthier et al. (2002) published a study performing ligation of both descending palatine arteries (in setting of Le Fort osteotomies); subsequent colored latex injection demonstrated perfusion of the hard palate mucosa via anastomoses between the greater palatine and ascending palatine arteries through the lesser palatine arteries and another soft palate collateral from ascending pharyngeal artery [6].

This study demonstrates the existence of vascular anastomoses between hard and soft palates and confirms that the section of the vascular pedicle of the flap is not necessarily related to flap necrosis [6].

In order to guarantee the blood supply of the hard palate through this anastomosis, the vascular connection should be preserved and the surgical dissection of the soft palate should be limited if the greater palatine artery is sectioned.

These findings support the concept described by Wardill and Dorrance in their techniques which include the section of both greater palatine pedicles in order to obtain proper length and closure of the palate with success, without any report of palatal necrosis in their group of patients [22, 23].

**Figure 8:**
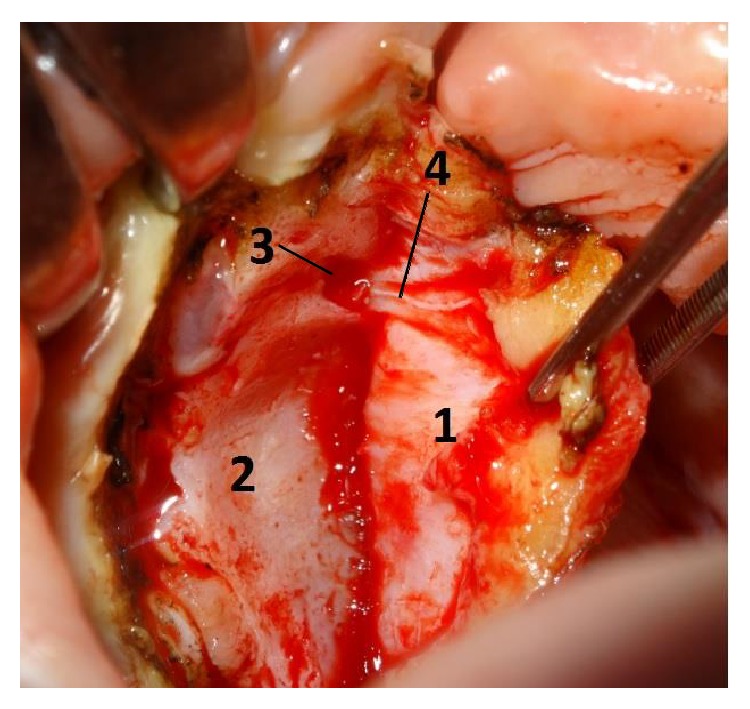
Anterior anastomosis of the palate. 1: mucoperiosteal palatal flap; 2: hard palate. 3: anterior palatine vessels and nerve coming through the anterior palatine foramen.

Traditional anatomical descriptions in noncleft humans consider the presence of an anterior palatine artery (from the sphenopalatine artery coming through the incisive foramen) and a vascular connection with the greater palatine artery, and this anastomosis has been observed only in unilateral cleft palates (noncleft side) and some incomplete cleft palates [5, 18] (Figure 8).

Maher in 1977 [18] developed an anatomical study with arteriographic examination in three human fetuses with cleft palate, based on Spriestersbach's theory who said that the aberrant craniofacial morphogenesis implies commensurate aberrant vascular supply [24].

During our surgical experience, we observe duplication, malposition, hypoplasia, and absence of the greater palatine foramen.

Vascular hypoplasia or absence is more common to observe in bilateral and incomplete cleft palates. Almost all cases of palatal necrosis evaluated during our practice belong to these types of cleft palates and seem to be the most probably related factor to the mucoperiosteal flap necrosis.

The growth of the nasal septum (vomer) outstrips the growth of other skeletal and soft tissues in the midface to such an extent that it is the pacemaker for growth of the face and anterior portion of the skull [25, 26].

The abnormal development of the nasal septum and nonattachment to the maxilla can be observed in bilateral and isolated (included submucous type) cleft palate [27, 28].

The role of this variations in the development of palatal flap necrosis remains unclear actually.

## 3. Etiology

Different etiologies have been described for the development of large defects after cleft palate repair like tension of the wound closure related to the surgeon's performance and cleft width, infection, and hematoma formation; however, it appears that necrosis of the mucoperiosteal flap is the most common cause of this complication [29].

The precise pathophysiologic events occurring in a failing flap are not totally understood.

To date there have been no major directly investigating the pathophysiology of conventional palatal mucoperiosteal flap failure after palatoplasty.

Palatal flap necrosis can be attributed to different causes.

These include mainly local causes (compression, tension, stretching, or section of the pedicle, vascular thrombosis, bleeding and hematoma, and surgical damage during the intervention).

In most cases, these causes can be minimized by careful perioperative management.

The association between use of local anesthesia with epinephrine and palatal flap necrosis was not studied yet; however, the use of lidocaine with epinephrine used before the surgery was found to have no harmful effect on the survival of nondelayed skin flaps.

This was the conclusion of an experimental study developed by Reinisch and Myers [30].

Compression of the pedicle is not a common event after palatoplasty.

Orientation of the greater palatine foramen and limited medial mobilization of the pedicle during the surgery make the compression of the greater palatine vessels difficult.

However, 2 factors may cause the compression of the palatal flap's pedicle: the use of islanded flaps and the severity of the cleft.

The utilization of islanded flaps, because of the section of the vascular anastomoses and the compression of the vascular pedicle over the greater palatine foramen due to the extended mobilization of the islanded flap.

Furlow [31] described before a relation between the use of island mucoperiosteal flap in association with his technique. He had 2 cases of flap necrosis in 100 operated cases.

Severe forms of cleft palate may affect the palatal flap's pedicle because of the tension and compression of the pedicle observed in these cases due to the extended mobilization of the flaps.

Wider cleft palates usually require extended dissection of the palate, mobilization of monopedicled flaps (based on hypoplastic vessels without additional blood supply from peripheral anastomoses), and surgical closure under some stretching and tension.

A study developed by Kuwahara and Yoshimoto found that older children and adults are more likely to develop hard palate mucoso-periosteal flap necrosis than infants in an investigation of 26 cleft palates in 13 patients aged 15 years or older that revealed that a number of anatomical differences were found when compared with infants [32].

Of these, abnormal bone protrusion appeared to be a factor that produced vascular compression and flap necrosis.

Stretching of a palatal mucoperiosteal pedicle flap also stretches vessels contained within it causing narrowing of their lumina and possible vascular occlusion and/or thrombosis.

The surgical injury of the pedicle (partial or total) is a rare event during the cleft palate repair. Its role in developing of palatal flap necrosis is not well studied.

Section of the vascular pedicle during the surgery has been associated with necrosis of the mucoperiosteal flap by some authors [2, 4].

However some authors concluded (based on previous observations) that the involuntary section of the greater palatine artery during the surgery is not necessarily in relation with flap necrosis [18, 20].

Controversy exists regarding the possible role of the artery's injury since authors like Dorrance and Wardill used the ligation of the vascular pedicle as a regular procedure during their surgical techniques for primary cleft palate repair without flap necrosis [22, 23].

This situation would be explained because of the vascular anastomoses between the greater palatine artery and the ascending palatine artery mentioned before.

Abnormal development of palatal vessels associated with the tissue's hypoplasia seems to be a probably related factor to the mucoperiosteal flap necrosis.

The growth of the nasal septum (vomer) outstrips the growth of other skeletal and soft tissues in the midface to such an extent that it is the pacemaker for growth of the face and anterior portion of the skull.

The abnormal development of the nasal septum and nonattachment to the maxilla can be observed in bilateral and isolated (included submucous type) cleft palates [27, 28].

Palatal flap necrosis is more common in these types of palatal clefts [1–3].

Other considerations related to the surgical technique and surgeon's performance are sutures too tightly secured and stitches inadvertently placed around the primary nutritional sources of a flap causing strangulation necrosis.

In addition, excessive manipulation of the pedicle may alter its blood supply causing ischemia and necrosis or atrophy.

Infection is a serious complication after palatoplasty because it may progress to flap necrosis [33, 34].

Ischemic or necrotic flaps may become infected secondarily and this condition is more commonly observed.

The extension of tissue necrosis may be increased by the presence of infection.

Zhang et al. have reported 9 cases of wound infection after cleft palate repair in 2100 patients [33] and Frolova et al. have found 13 cases with infectious inflammations of the wound from a sample of 153 babies after cleft palate surgery [34].

Primary infection of the surgical wound is rare and may be in relation to patient's immunodeficiency (mainly associated with severe chronic malnutrition).

Palate necrosis as a consequence of a palate infection has been reported by Sancho et al. in a 6-month-old child who presents this complication in relation to a suppurative medical otitis that involved hard and soft palates. The culture was positive for* Pseudomona aeruginosa*  [35].

Careful examination and diagnosis of middle ear status and blood tests are recommended before cleft palate surgery in order to avoid this complication.

A study published by Maine et al. [36] observed a probable relation between the development of palatal fistulas after cleft palate repair and nutritional status of the patients; however, this association is not well demonstrated yet.

Additional studies are required in order to establish the association between the nutritional status and the development of fistulas or palatal necrosis.

A prospective study to evaluate possible pathogenic organisms associated with wound complications in the form of wound infections, wound breakdown, and the formation of oronasal fistulas was performed by Mÿburgh and Bütow and found that a group of organisms that originated from the colon/perineum is mostly associated with these postoperative complications [37].

The antibiotic resistance profile showed a high resistance to antibiotics such as ampicillin, amoxicillin-clavulanic acid, and first- and second-generation cephalosporins.

Frolova et al. [34] have found (on day 3 after the operation) Gram-negative Bacilli isolated from the majority of patients with postoperative wound infection coursing in the presence of marked dysbacteriosis.

Chuo and Timmons [38] in a retrospective study found that children with unrepaired cleft lip and palate have a significant risk of carrying* S. aureus* and a small risk of carrying beta-hemolytic* Streptococci*.

However, colonization by* S. aureus* decreased significantly following surgical repair of the cleft lip and palate [39].

A prospective study published by Cocco et al. [40] observed a direct relation between palatal dehiscence and the presence of beta-hemolytic* Streptococci* and recommend a screening for* Streptococci* prior to surgery routinely.

All these studies did not evaluate association between pathogenic organisms and palatal necrosis.

Finally, the association between bleeding (hematoma) and mucoperiosteal flap necrosis is not well establish and additional studies are required.

Hematoma related necrosis of palatal flaps does not occur only because of the internal pressure. A toxic effect of the mass of blood on skin flaps has been demonstrated by Mulliken and Healey in an experimental rat model [41].

The role of bleeding as risk factor for flap necrosis could be related to the minimal incision technique because of the absence of lateral raw surfaces.

## 4. Diagnosis

This complication is characterized by early signs after palatoplasty which are change in a flap color (initially pale and then dark) associated with bad odor during the first days.

Signs of infection may be present and include swelling of the palate, irritability, raised temperature, and loss of appetite.

**Figure 9:**
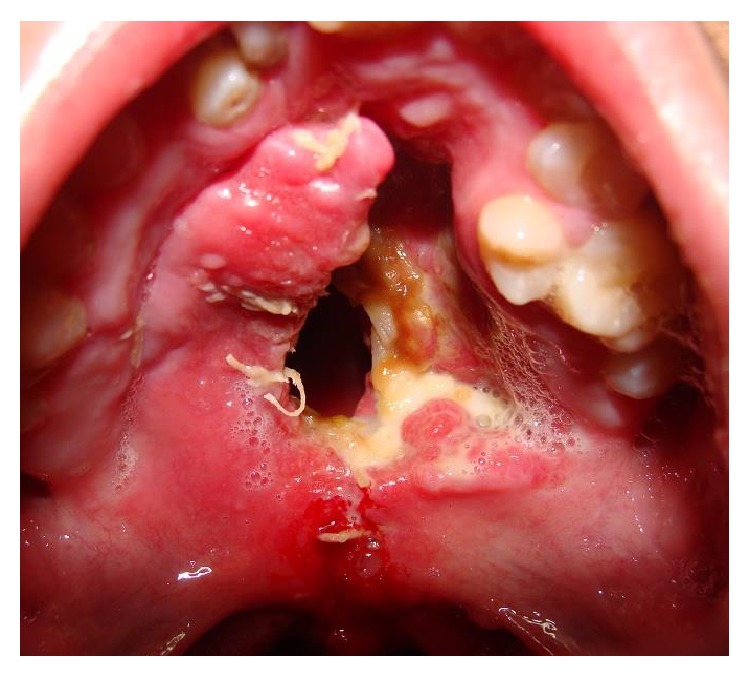
Twenty-one-year-old patient with incomplete cleft palate. After one week the patient returns and the repaired cleft palate showed necrotic tissue and dehiscence of the palate closure.

After 5 to 7 days, dehiscence of the surgical wound closure, loss of necrotic tissue, and some bleeding appear (Figure 9).

**Figure 10:**
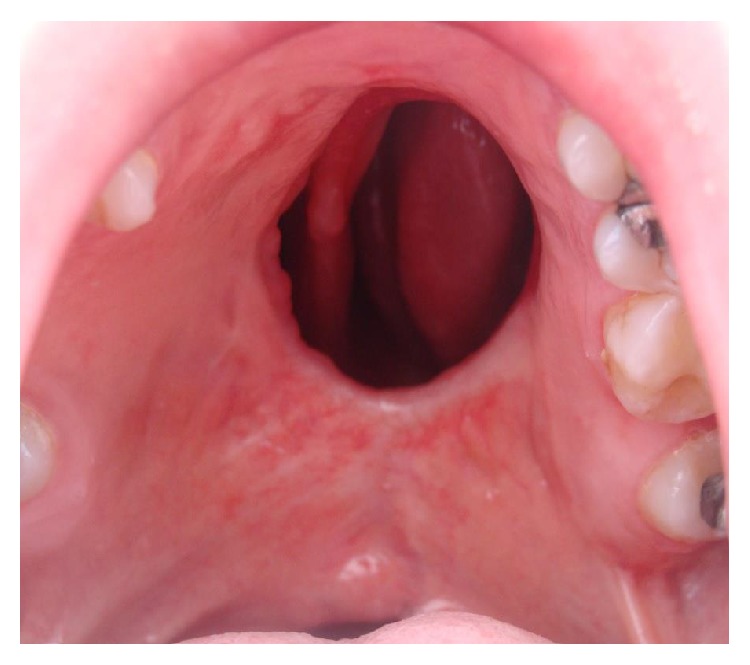
Large fistula after cleft palate repair in a 28-year-old patient with incomplete cleft palate.

**Figure 11:**
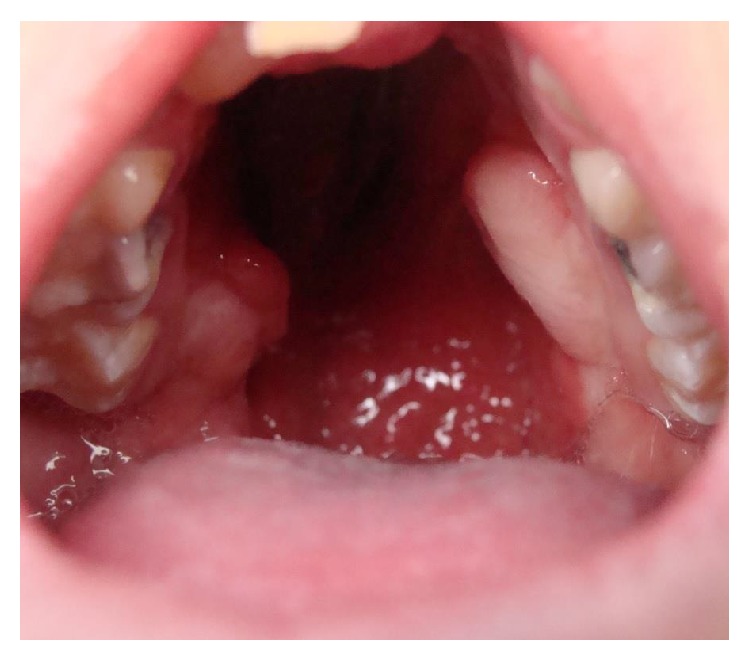
Large defect after cleft palate repair in a 12-year-old patient with bilateral cleft palate. The surgery developed bilateral palatal flap necrosis and the wound healed leaving a large defect in the hard and soft palate.

Then, the exposed palatal bone is resorbed leaving a defect which is characterized by large dehiscence or fistulas (bigger than the initial congenital defect) (Figures 10 and 11).

Blood tests are necessary in order to establish the diagnostic of infection associated with the necrotic tissue and the requirement of antibiotics.

## 5. Prevention

In most cases, causes of flap necrosis can be minimized by careful preoperative planning and prevention is possible.

Nutritional status of the patient, associated diseases as middle ear infections, knowledge of the vascular anatomy of the palate, and type of the cleft should be considered during the preoperatory evaluation.

Based on the reviewed information we may consider the following recommendations in order to prevent palatal flap necrosis.

Cleft palate's degree of hypoplasia should be estimated before the surgery in order to design a proper surgical planning and prevent nondesirable outcomes.

Most of the reported cases of palatal necrosis are clefts with more tissue's hypoplasia.

We design a predicting scale for mucoperiosteal flap necrosis after primary palatoplasty to evaluate cleft palate's degree of hypoplasia.

This scale evaluates degree of hypoplasia and is based on the following items.

*(a) Type of Cleft*. It is based on Veau's classification for cleft palate deformity [42]:

1. soft palate (score: 1),
2. soft and hard palates (score: 4),
3. unilateral soft and hard palates (score: 2),
4. bilateral soft and hard palates (score: 4).

*(b) Index of the Cleft Palate*. It is based on the palatal index description for cleft palate deformity (proportion between the width of the cleft (cleft's severity) and the summary of the width of the two palatal segments (tissue deficiency)) measured at the level of the hard and soft palates junction [43]:

1. mild index: less than 0.2 (score: 1),
2. moderate index: 0.2 to 0.4 (score: 2),
3. severe index: greater than 0.4 (score: 4).

*(c) Length of Soft Palate*. It is based on Randall's classification for cleft palate deformity [44].

1. Uvula reaches the posterior pharyngeal wall (score: 1).
2. Uvula reaches the posterior half of the adenoid pad (score: 2).
3. Uvula is located at the anterior half of the adenoid pad (score: 3).
4. Uvula is located anteriorly to the adenoid pad (score: 4).

### 5.1. Grading Scale Score

 Low risk is total score 3–5.
 Moderate risk is total score 6–8.
 High risk is total score 9–12.

Based on the cleft palate's degree of hypoplasia scale we may propose the following surgical protocol:

 (a) *mild (risk score 3–5)*
   
*unipedicled flaps (two-flap palatoplasty),*
 (b) *moderate (risk score 6–8)*
   
*bipedicled flaps (Von Langenbeck technique),*
 (c) *severe (risk score 9–12)*
   
*soft palate closure + vomer flap (delayed hard palate closure).*

This scale has not been validated before and will be studied in the near future.

Surgical design should be based on the anatomical considerations described before and the surgical technique should be carefully selected based on the type of repair, type of cleft, and its severity.

During cleft palate surgery all the vascular anastomoses are sectioned using flaps based in one pedicle (two-flap technique) (Figures 5 to 7).

This technique has an increased risk of palatal flap necrosis and should be avoided in cleft palates with hypoplastic vessels (bilateral and incomplete cleft palates).

Unipedicled flaps are used in our program only in the noncleft side of unilateral cleft palates because the arteries are well developed.

Anterior vascular anastomoses (branches coming from infraorbital, alveolar, and superior labial arteries) are preserved using the Von Langenbeck method.

However, these vascular connections may be affected by the lip surgery previously done in special branches from infraorbital and superior labial arteries. The labioalveolar sulcus incision during cheiloplasty should be limited in order to preserve these vessels.

This technique is recommended in cleft palates with hypoplastic vessels (incomplete and bilateral cleft palates).

**Figure 12:**
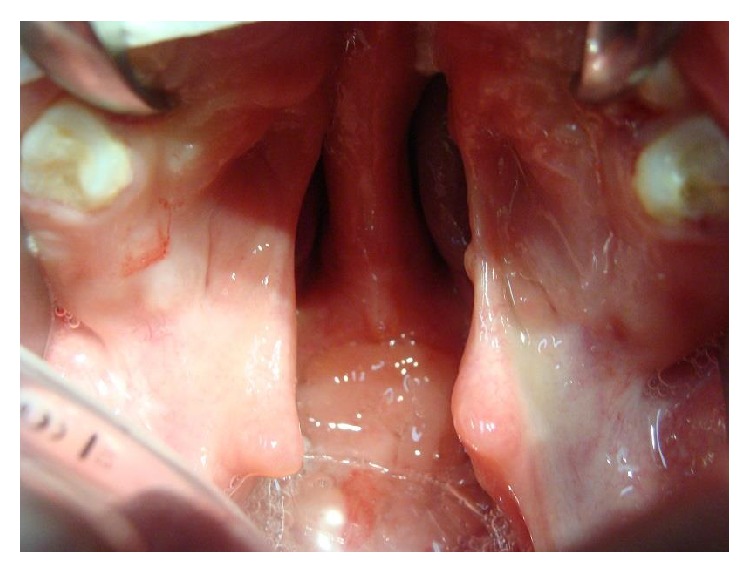
A one-year-old girl with a history of bilateral cleft lip and palate. The cleft palate was classified as Veau 4, Randall 4, and severe palatal index (0.48) with high risk score for palatal necrosis.

**Figure 13:**
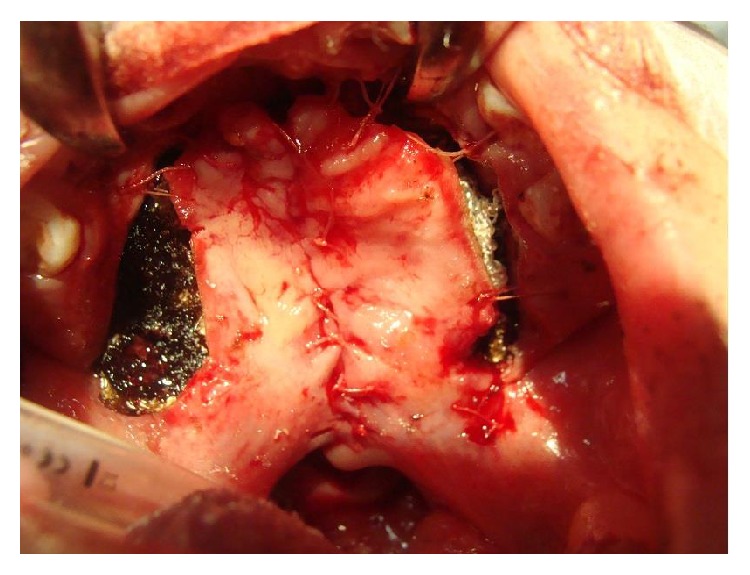
The cleft palate was closed using the two-flap palatoplasty at on year old.

**Figure 14:**
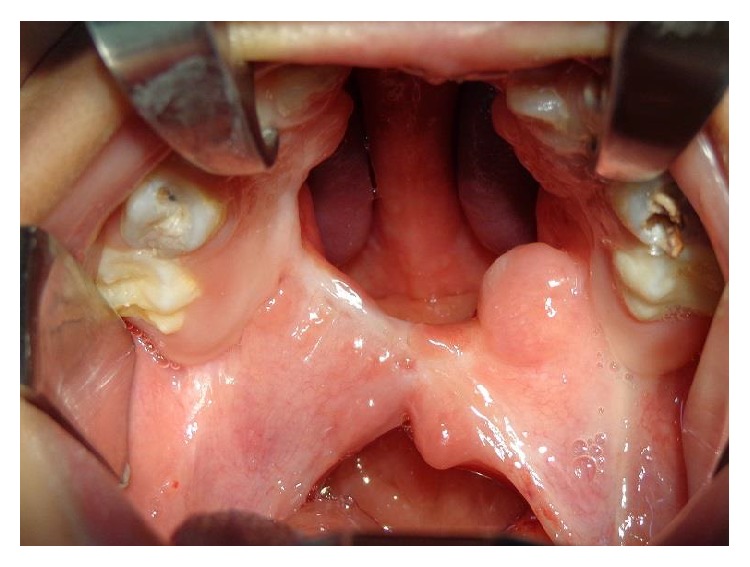
The surgery developed bilateral palatal flap necrosis and the wound healed leaving a large defect in the hard and soft palate.

Severe forms of incomplete and bilateral cleft palate require a careful surgical design to prevent this devastating complication (Figures 12, 13, and 14).

Bipedicled flaps (based on Von Langenbeck's concept) are recommended for these types of clefts. In severe bilateral cleft palates, we use the delayed hard palate closure without elevation of mucoperiosteal flaps.

Variations in palatal arterial distribution cannot be determined before surgery.

However, changes in position and hypoplasia or absence of these vessels can be identified during the surgery if the procedure is performed with caution avoiding damage of these structures and taking decisions to preserve vascular anastomoses.

Injury of the greater palatine artery is not necessarily related to flap necrosis; however, this situation requires a special management in order to prevent this complication.

Our protocol under this scenario includes first the compression of the greater palatine foramen using some resorbable material in order to control the hemorrhage.

Then the surgical dissection should be stopped at the side of the injured vessel preserving the vascular anastomosis between the mucoperiosteal flap and the soft palate.

The cleft palate surgery can be continued doing an extended dissection of the tissues in the opposite side in order to obtain a surgical closure with minimal tension.

Similar proceeding is recommended in case of injury of the greater palatine vessels with the cautery or surgical needle.

Finally, in relation to the wound infection. Frolova et al. suggest a method for wound infection prevention in uranoplasty, consisting in irrigation of the operative wound with acilact suspension (a biopreparation) and shortening of antibiotic prevention course to just 48–72 [34].

However, this method required additional studies to evaluate its efficacy.

The data obtained in a study developed by Savenkova et al. [45] show that the development of intercurrent diseases and postoperative complications (not specifically palatal necrosis) can be prevented by the parenteral application of cephalosporins of the III and IV generations as well as by oral administration of cefixime and protected aminopenicillins.

This antibiotic prophylaxis protocol requires scientific validation.

## 6. Management

Initial management of this complication may require surgical debridement of the necrotic tissue; however, most of the patients presented for follow-up few days after surgery had an autolytic debridement of the necrotic flap and irrigation of the wound and antibiotics during 5 days are only necessary to prevent any infection (Figure 9).

As some bleeding may be associated, if this is moderate or severe, reoperative hemostasis is required in surgical room.

Treatment of the sequels especially when the defects are wide and scarred is a challenge for both patients and plastic surgeons, with high rate of recurrence.

Large defects after cleft palate repair produce various symptoms, including regurgitation of fluid into the nasal cavity, hearing loss, and velopharyngeal insufficiency.

In these cases, the palatal tissue around the fistula can be quite scarred and in short supply.

A variety of reconstructive options are commonly employed, using local and distant flaps or combination of both.

**Figure 15:**
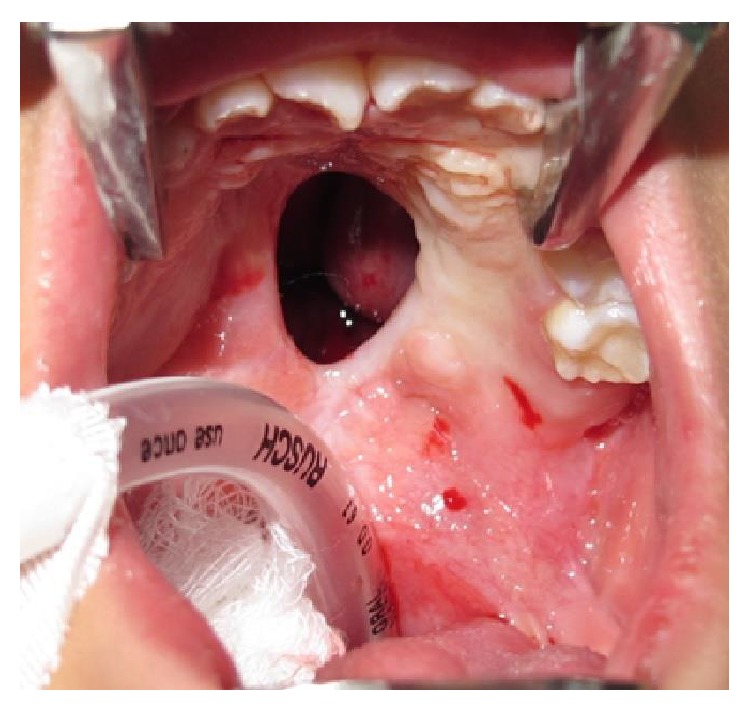
Nine-year-old patient with diagnosis of incomplete cleft palate and a severe fistula after cleft palate repair complicated with flap necrosis.

**Figure 16:**
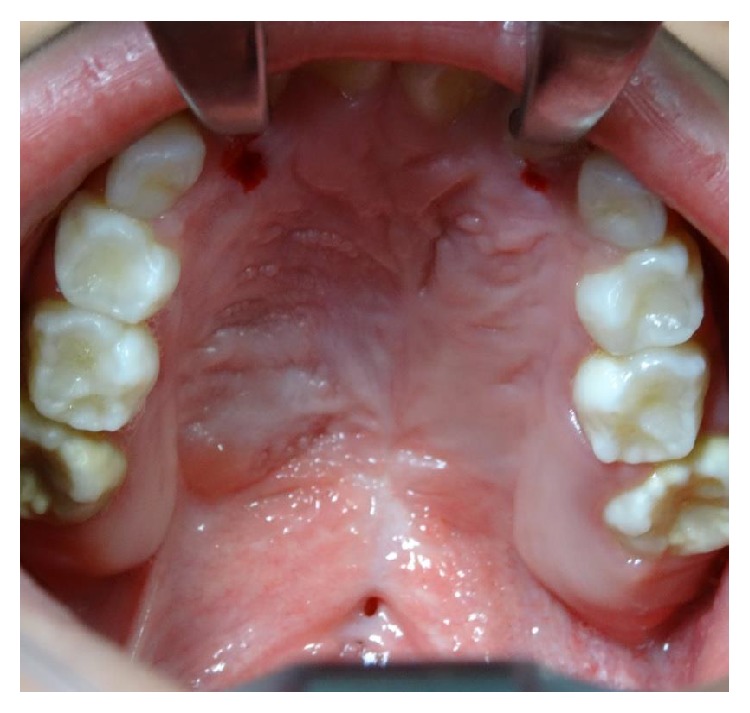
Postoperative view of the palate after one year. The defect was closed using two mucoperiosteal flaps.

The first option in our protocol of management is the use of local flaps (Figures 15 and 16).

The availability of healthy tissue from palatal mucosa should be evaluated and identification of greater palatal artery patency using Doppler is necessary.

However, at times the site and the size of the fistula make use of local flaps for its repair a remote possibility.

The combination of buccal mucosa and buccinator muscle as an axial myomucosal flap based on the facial artery has been described by Pribaz et al. [46].

This flap consists of mucosa, submucosa, part of the buccinator and orbicularis muscles, and the facial artery with its venous plexus.

**Figure 17:**
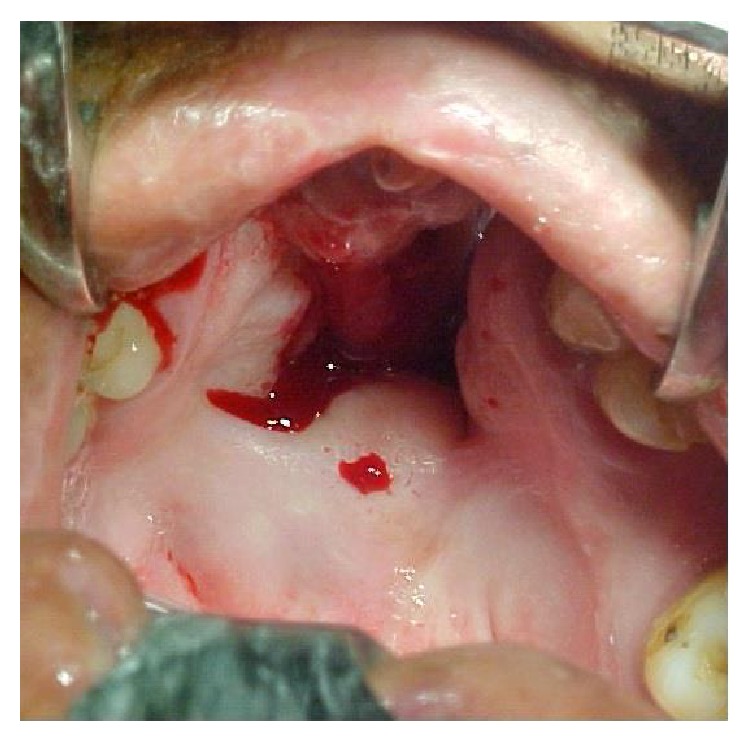
Severe anterior fistula after cleft palate repair in a 32-year-old patient with bilateral cleft palate. Surgery was complicated with distal flap necrosis.

**Figure 18:**
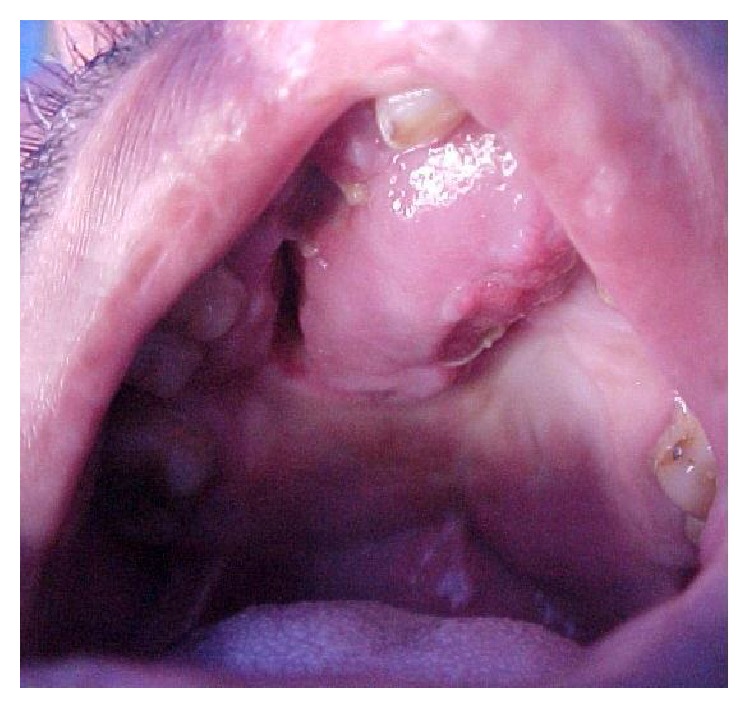
The fistula was closed using the FAMM flap.

This is known as the facial artery musculomucosal (FAMM) flap and is one of the most used flaps for intraoral defects (Figures 17 and 18).

Partial flap necrosis has been described in 18% of cases using this flap [47].

Some described limitations of this flap are as follows: surgical elevation can be technically challenging and its pedicle may interfere with the dental occlusion and eruption of permanent molars. Close to 26% of cases required further surgery to divide the bridge segment of the flap usually after 3 weeks [48].

Bite block may be required postoperatively in order to prevent an injury of the flap.

The author of this paper described a variation of this flap including an island of skin (named as FAMMC (facial artery myomucosal cutaneous) flap) to be used when the amount of nasal mucosa is not enough [49].

We observed partial necrosis in one case (8.3%) and one dehiscence (8.3%) using this flap.

Two-layer method of fistula repair is recommended in any technique to avoid recurrences.

In conventional fistula repair, the fistula margins are dissected 2 to 3 mm around the fistula as a turndown flap from oral mucosa closing the nasal layer of the palate.

However, this procedure is not adequate to close the nasal layer in larger fistulas.

Use of FAMMC flap and utilization of pharyngeal flaps or mucosal grafts are indicated in these cases [49–51].

Main limitations for facial artery flaps are requirement of an open dental arch for passage (anteriorly based type), width of the flap limited to 1.5 to 2 cm, and the inclusion of facial muscles, which could interfere with speech development.

In addition, these local axial flaps are hampered by a need for scrupulous postoperative patient compliance.

The pedicle buccal fat pad flap is another option as combined method when the nasal mucosa is repaired and there is deficiency of tissues from oral mucosa [52].

Similar use has been described for amniotic membrane allograft and a cellular dermal matrix [53, 54].

**Figure 19:**
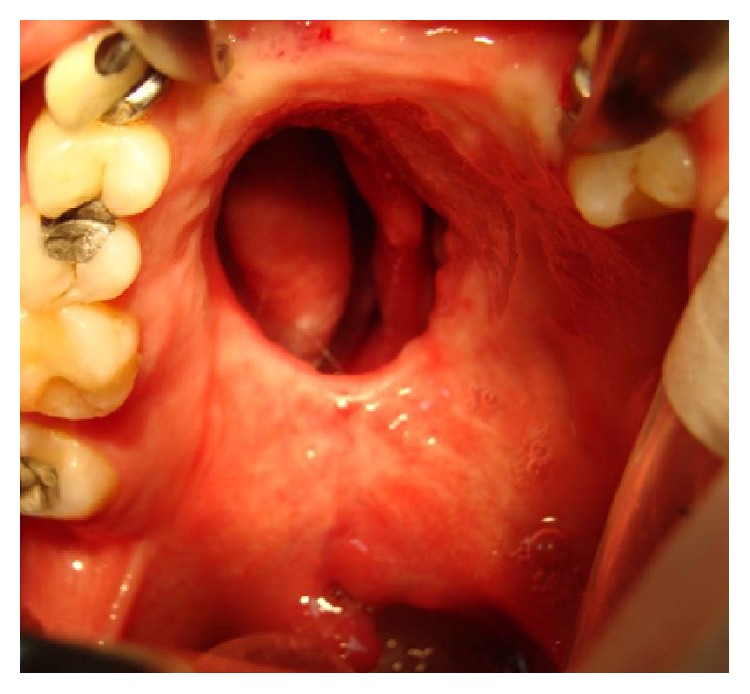
Large fistula after cleft palate repair in a 28-year-old patient with incomplete cleft palate. The surgery developed extensive flap necrosis and the wound healed leaving a large defect in the palate.

**Figure 20:**
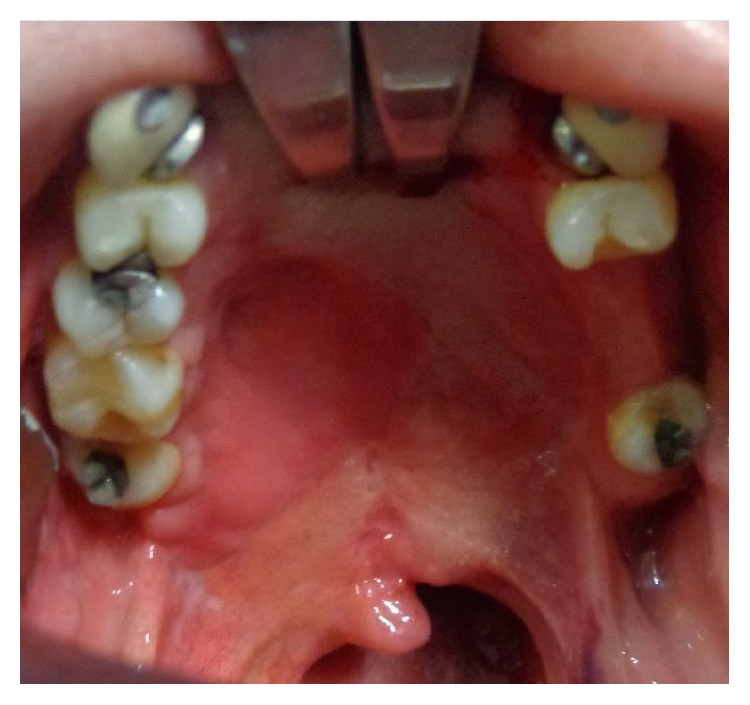
The defect was repaired using a combination of local mucoperiosteal flap and FAMM flap.

Combination of local flaps and facial artery flaps can is recommended when the defect is too large (Figures 19 and 20).

Use of temporoparietal-galeal flap and temporalis muscle flap has been described for palatal fistula repair too [55, 56].

This technique has been described as being able to cover palatal defects; however, it usually leaves a temporal hollow as a donor-site deformity and in children might not be sufficiently developed for transposition [57].

In addition, for adequate transposition and sufficient length, an osteotomy of the zygomatic arch might be necessary, with the dissection procedure endangering the frontal arch of the facial nerve.

Tongue flaps were introduced for intraoral reconstruction by Lexer in 1909 [58].

The excellent vascularity and the large amount of tissue that tongue flaps provide have rendered the flaps appropriate for the repair of large fistulas in operated cleft palates.

**Figure 21:**
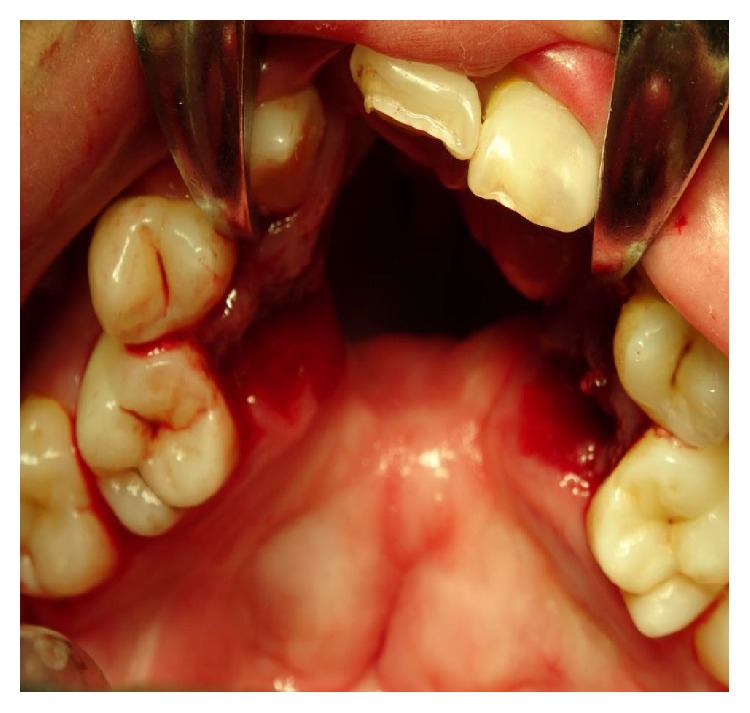
Fifteen-year-old patient with bilateral cleft lip and palate who has a large anterior palatal fistula after distal mucoperiosteal flap necrosis.

**Figure 22:**
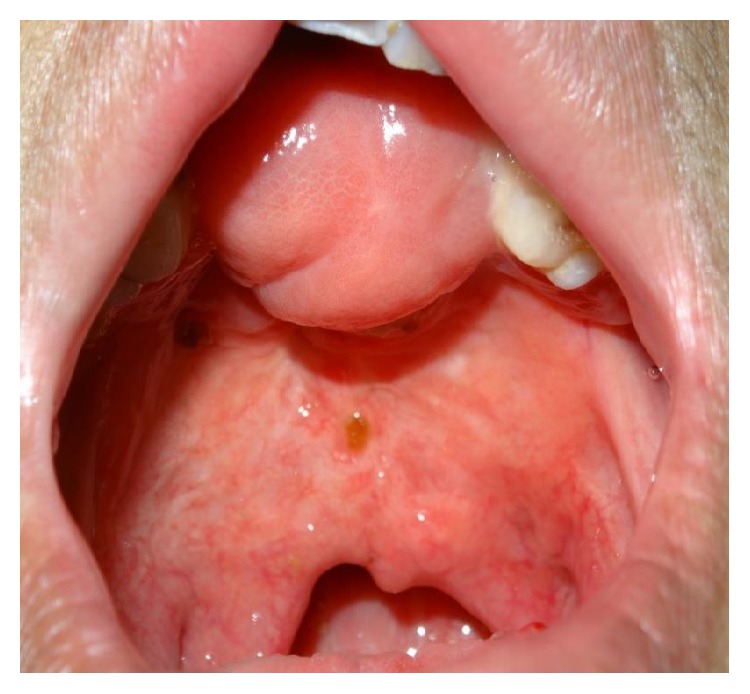
Fistula was closed using an anteriorly based tongue flap. After 2 weeks the flap was divided.

The central position in the floor of the mouth, mobility, and the diversity of positioning the flaps make it a method of choice for closure of anterior palatal fistula especially (Figures 21 and 22).

Complications include hematoma, sloughing, epistaxis, dehiscence, loss of tongue taste and sensation, narrowing of the tongue, and flap necrosis with recurrence of fistulas [59–61].

Described intraoral flaps are actually the standard of care; however, the donor-site morbidity, multistage operative protocols, and the use of nasogastric tube for patient's feeding during some days required for many of these flaps make them less than ideal.

Microvascular tissue transfer may be indicated for more severe cases with large defects, extensive scar tissue around the fistula, and repeated failure of conventional methods.

With experienced hands and proper teamwork, free-tissue transfer has achieved a success rate of 95 percent [62].

First dorsal metatarsal artery dorsalis pedis flap, angular scapular flap, radial forearm flap, anterolateral thigh flap, and the temporal parietal flap were described with this purpose [62–66].

Use of free flaps requires competence in microsurgery, longer operative time, and prolonged hospitalization.

It may also lead to donor site morbidity and esthetically unsatisfactory results [62].

Donor site morbidity should be well considered for flap selection.

Use of tissue expanders has been described in palatal fistula repair; however, its utilization is not widespread and additional studies are required [67, 68].

Use of platelet rich plasma mixed with autologous bone graft seems to be an effective, safe, and low-cost technique for the closure of recurrent cleft palate fistulas [69].

The rehabilitation using obturator prosthesis is an option to surgical treatments [70].

In cases where the surgical treatment is contraindicated, the prosthetic rehabilitation becomes a definitive treatment [71, 72].

However, patients using obturator prostheses often present complications as ulcerations and stomatitis related to* Candida albicans* [73].

The presence of large oronasal communications alters the normal oral environment and different results are expected in this situation.

Reconstruction of the velopharyngeal sphincter is usually required in these cases.

## 7. Summary

Palatal necrosis after cleft palate repair is a rare but significant problem.

The extent of functional impairment is great and has psychological, social, and developmental consequences. Sequels affect feeding and speech of these patients.

Vascular anatomical variations including hypoplasia or absence of greater palatine vessels, injury of the pedicle, cleft type, surgeon's performance, and used surgical technique may be in relation to this complication.

Other risk factors such as nutritional status, associated anomalies, and concomitant infection should be evaluated and further prospective studies are necessary.

In most cases, causes of flap necrosis can be minimized by careful preoperative planning and prevention is possible.

Surgical techniques used for treatment of the sequels should be carefully selected based on sized and location of the defect and patient's condition.

## Figures and Tables

**Table 1:** Analysis of patients with cleft palate operated by three centers in Lima, Peru, who developed palatal flap necrosis 1994–2013.

	Center A	Center B		Center C	Total
	Case 1	Case 2	Case 3	Case 4	
Age^*^	1	2	24	1	
Sex					
Male	1	0	0	0	1
Female	0	1	1	1	3
Type of cleft					
Veau I	0	0	0	0	0
Veau II	0	1	1	0	2
Veau III	0	0	0	0	0
Veau IV	1	0	0	1	2
Prevalence	155/1 (0.64%)	325/2 (0.61%)		694/1 (0.14%)	1,174/4 (0.34%)

^*^Age at the time of the surgery.
